# Patient-Centered Treatment Outcomes with Full-Arch PEEK Rehabilitation Supported on Four Immediate or Conventionally Loaded Implants. A Randomized Clinical Trial

**DOI:** 10.3390/jcm10194589

**Published:** 2021-10-05

**Authors:** Javier Montero, Yasmina Guadilla, Javier Flores, Beatriz Pardal-Peláez, Norberto Quispe-López, Cristina Gómez-Polo, Abraham Dib

**Affiliations:** Department of Surgery, Faculty of Medicine, University of Salamanca, 37007 Salamanca, Spain; yguadilla@usal.es (Y.G.); j.flores@usal.es (J.F.); bpardal@usal.es (B.P.-P.); norberto_quispe@usal.es (N.Q.-L.); crisgodent@usal.es (C.G.-P.); ibrahimdib@usal.es (A.D.)

**Keywords:** patient-reported outcomes, prosthodontics, dental implant, mastication, hybrid denture, immediate loading

## Abstract

This study aims to assess the treatment outcomes (functional and subjective) of full-arch fixed hybrid rehabilitations made of PEEK (poly-ether-ether-ketone) with milled crowns of nano-filled composite (NFC) supported on four to six implants. In this randomized clinical trial, 34 edentate patients in the upper and/or the lower jaws were treated with the fixed hybrid dentures. In 16 patients (47.1% of the sample), the implants were loaded immediately (IL) by means of a provisional fixed rehabilitation made of PMMA (polymethylmethacrylate) screwed on Multi-Unit (MU) abutments connected after emplacement of the implant; however, in the counterparts (*n* = 18) these MU abutments were covered by healing caps and were left unloaded during two months (conventional loading protocol—CL), when all patients received a fixed hybrid PEEK-NFC rehabilitation on the upper and/or the lower jaw. Treatment outcomes were assessed 12 months after prostheses delivery. Functional outcomes were calculated according to masticatory performance, estimated by mixing ability tests of two colored chewing gums after ten chewing strokes, by the occlusal force/area recorded by pressure-sensitive sheets, and by electromyography of masseters and temporal muscles at maximum biteforce. The subjective outcomes of the treatment were assessed using both the oral satisfaction scale (visual analog scale) and the Spanish version of the Oral Health Impact Profile (OHIP-20). The findings of the present study showed that treatment with fixed PEEK-NFC hybrid prostheses significantly improved the masticatory performance, bite force, occlusal pattern, quality of life, and satisfaction, with the IL group being those with significantly higher occlusal bite forces and greater satisfaction in comparison with CL group. It should be concluded that PEEK-NFC hybrid prostheses can improve several patient-centered outcomes and that loading protocol significantly affects the patient’s self-rated satisfaction.

## 1. Introduction

The natural evolution of the most prevalent dental diseases (caries and periodontal disease) contributes to dental loss, usually after ablative therapeutic intervention. The progressive loss of teeth leads people to become edentulous. Of the Spanish elderly (65–74 years), 7.3% (CI 95% 4.4–10.2%) are fully edentulous and wear complete conventional dentures [1] and usually suffer from oral pain, functional problems [2], and poor quality of life [3]. However, until more than 30 years ago conventional complete dentures were the only therapeutic resource for replacing missing teeth in fully edentulous patients. Since then, new technical approaches, abutments, and materials have refined the use of dental implants as retention elements of dental prostheses with the aim of improving the therapeutic effect and its predictability [4,5]. The indication of either implant-retained or implant-supported prostheses was born and is still especially indicated in the edentulous mandible, since the area of support of this jaw is much lower than that of the maxilla (which takes advantage of using the hard palate as retention area), being, therefore, the most uncomfortable place to wear conventional dentures. That is why overdentures retained on two implants are now considered the standard of care for mandibular edentulism [6].

Both implant-retained overdentures (IO) and implant-supported fixed hybrid rehabilitations (ISFR) can provide adequate support for the perioral soft tissues, resulting in a better natural-looking smile among edentates, whose dental loss was accompanied by a moderate–severe reduction in the residual alveolar ridge volume. For such purposes, overdentures are more cost-effective than fixed restorations because they need fewer implants and components [7]. In contrast, fixed restorations provide higher maximum occlusal force and better masticatory performance than overdentures [8] as objective outcomes assessments. Moreover, despite no difference being found in clinical terms (implant survival and marginal bone loss) between IO and ISFR [9], the last option gives better quality of life and satisfaction to patients, mainly for pain-, comfort- and function-related domains [9,10]. According to a recent meta-analysis performed by Borges et al. [9], there is a lack of studies documenting patient-reported outcome measures (PROM) for implant prosthodontics, in particular, for oral health-related quality of life (OHRQoL) and satisfaction. Similarly, most of the evidence regarding the treatment outcomes of the ISFR comes from either layered ceramic fused to metal or resin-veneered metal frameworks [11] (usually gold alloys, titanium, or Cobalt–chromium) [12], and there is a lack of studies focusing on ISFR made of innovative materials such as PEEK (poly-ether-ether-ketone) veneered with nano-filled composite (NFC) [13]. In the field of dentistry, the possibility of computer-aided design/computer-aided manufacturing (CAD/CAM) together with its biocompatibility and shock absorbing features [14] has enabled the increased use of PEEK as substructure material to be veneered with composite or acrylic resins. 

Hence there is a need for studying the impact of the PEEK-NFC hybrid prostheses on patients’ wellbeing and oral function, after replacing both the missing soft and hard tissues. Recent evidence suggests that hybrid polymer (PEEK—polymethylmethacrylate) acrylic resin prostheses supported by implants for full-arch rehabilitation may represent a valid treatment option for edentulous patients in the short term [15,16], but rehabilitation made of PEEK covered by composite instead of acrylic resin has not yet been evaluated to date.

The original protocol for rehabilitating implants is the conventional loading (CL) that requires a 3–6 month period of bone healing in which the implants are still not connected to the prostheses [4], and hence, patients are forced to maintain their previous poor oral performances during such period. That is the original rationale of establishing an immediate loading protocol (IL), which implies the application of functional loads on implants immediately after insertion in bone. It is widely known that IL may be as effective as CL if proper primary stability is feasible in the remaining bone [17], commonly assessed by insertion torque and ISQ values [18]. Moreover, from the functional and psychological points of view, IL is expected to be of greater benefit for the patient due to the immediate restoration of mastication, phonetics, and esthetics.

The purpose of the present study was to evaluate the functional and subjective outcomes of implant-supported full-arch hybrid rehabilitations made of PEEK-NFC for treating edentate patients depending on the loading protocol.

## 2. Materials and Methods

### 2.1. Study Design

This randomized clinical trial (RCT) was designed to make both cross-over and parallel comparisons. The former was carried out by comparing the intra-subject outcomes between baseline conditions and after the rehabilitation with the new PEEK_NFC). The latter was obtained by comparing outcomes of the PEEK_NFC with IL or CL protocol. The null hypothesis assumed that there would be no difference in either functional or subjective outcomes in any of the above-mentioned comparisons.

To effectively conceal the randomization sequence, we used sequentially numbered (odds for test, pair for controls) lots placed within sealed opaque envelopes that were shuffled. Later when all the implants were emplaced in the planned sites for a given patient, the assigned envelope was opened.

This research was conducted in accordance with the ethical principles of the World Medical Association Declaration of Helsinki, which has been previously approved by the Bioethics Committee of the University of Salamanca (Spain) (RUSAL_201500006834) and recently inscribed to ClinicalTrials.gov with the following identifier: NCT04930835. All patients provided written consent to participate in the study and undergo both the planned surgical and prosthetic interventions.

Calculation of the sample size of this study was based on our previous experience assessing the quality of life among edentulous patients with the Spanish version of the OHIP-20 [19]. It was then estimated that each group should be comprised of at least ten patients to detect differences in the means of five points with two-sided tests with a power of 80% and an α error of 0.05. However, to maintain enough statistical power for either bivariate or subsample comparisons, and also to compensate the sample attrition with follow-up, it was decided to oversample to, at least, fifteen subjects per group.

The study sample was recruited from totally or partially edentulous patients needing full-arch implant-supported rehabilitations attending the University Dental Clinic of the Faculty of Medicine at the University of Salamanca.

In our study, 34 patients received 210 implants for supporting the PEEK rehabilitations. All patients included were completely or partially edentulous (but with non-restorable standing teeth) in at least one jaw that wore conventional prostheses, with enough quantity/quality of bone to receive four implants with an insertion torque > 40 Ncm. Patients with evidence of systemic or psychic pathology that contraindicate the implant treatment were excluded.

### 2.2. Preoperative Assessments

A face-to-face interview between patient and dentist gathered both the sociodemographic and the subjective data. Afterward, functional data were collected by recording on pressure-sensitive colorimetric sheets (Dental PRESCALE, Fuji Photo Film Co, Tokyo, Japan) the occlusal contact area (mm^2^) and the maximal voluntary occlusal force (Nw), while registering the muscular activity by surface electromyography on both masseters and temporalis muscles (MYOMED_932 TM; Enraf-Nonius B.V., Rotterdam, The Netherlands).

In addition, the mastication was estimated objectively by calculating the mixing ability of two colored chewing gums (Smint Kiss 3, Chupa Chups SL, Barcelona, España) as reported by Montero et al. elsewhere [20].

**Figure 1:**
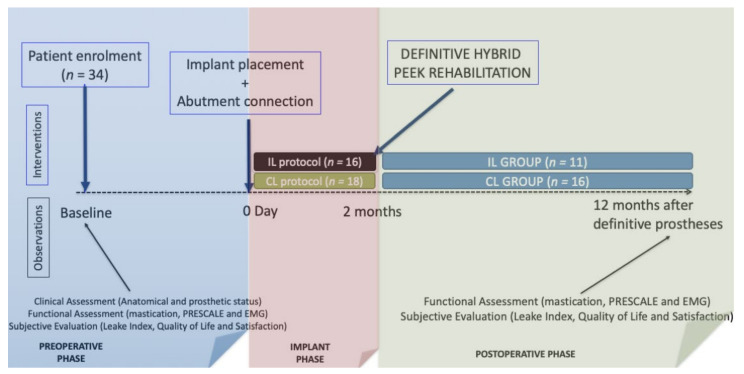
Diagram of the observations and interventions scheduled in the study.

The diagram of the observations and interventions carried out in this study is shown in Figure 1. In the pre-operative phase, patients were explored to record the baseline clinical, functional and subjective data. In this regard, the chewing ability was evaluated by registering the self-reported difficulty in chewing five target foods (apple, salads, meat, boiled vegetables, and carrots) according to the Leake Index [21]. Moreover, data regarding self-reported satisfaction on a visual analog scale [22] and the oral health-related quality of life according to the Spanish version of the OHIP-20 (Oral Health Impact Profile) [19] were also gathered. This OHIP-20 [19] is specifically useful to quantify the impact of oral conditions edentate across seven domains, i.e., functional limitation, pain, psychological discomfort, physical disability, psychological disability, social disability, and handicap. The frequency responses of the 20 items were coded numerically from 0 = never to 4 = very often as Likert scales. The total scores of the OHIP-20 were calculated by simply counting the number of items recorded as occasionally or more frequently (≥2). Hence, the OHIP-20 summary score ranged from 0 to 20.

### 2.3. Surgical and Prosthetic Treatment Protocol

On the day of the surgical intervention, all patients received local anesthesia for the emplacement of at least four dental implants distributed usually in the canine and molar regions, following standardized surgical protocols to insert implants (>40 Ncm) in a crestal position. Both the quality of bone and the gingival biotype covering such bone were clinically determined and then classified from D1 to D4 for bone [23], and thick (<2 mm), medium (1–2 mm), or thin (<1 mm) for gingiva after flap elevation.

**Figure 2:**
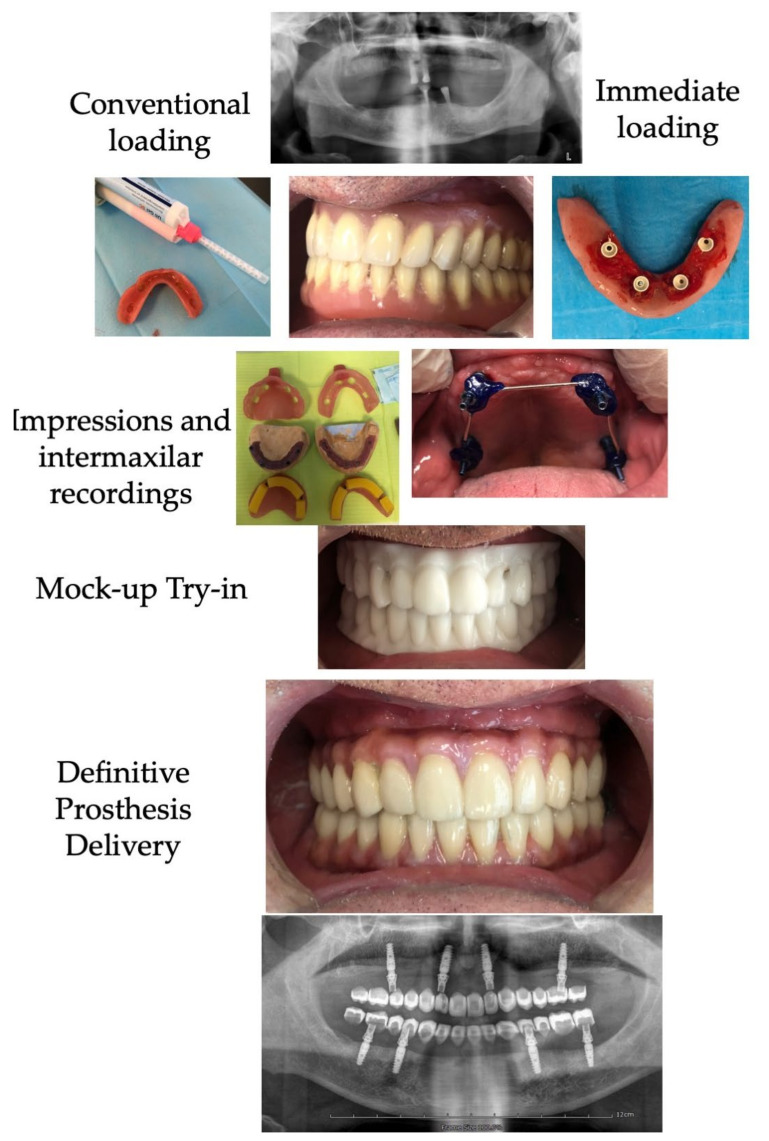
Diagram of the treatment steps for the rehabilitation with PEEK-NFC rehabilitation.

All the placed implants received multi-unit^®^ abutments which were connected to provisional fixed prostheses through PEEK transitional abutments by pink acrylic resin (Kooliner GC, Kortrijk, Belgium) (IL group), or to healing caps (CL group) that were covered by removable transitional prostheses (the precedent one in most cases) that had been strategically ground down by the internal surface in order to make room for the abutments, or by a transitional tooth-supported fixed prostheses. In summary, among IL patients, the implants exclusively supported the functional loadings immediately after implants, while among the CL group, either the remaining mucosa or transitional teeth were responsible for supporting the functional loads. Figure 2 shows the distinct treatment steps.

A mutually protected occlusion scheme was established in most cases, except when both arches were treated by CL in which case a properly bibalanced occlusion was adopted [24].

After two months, the protocol for the definitive prostheses construction was initiated as follows: after removing the provisional prostheses (IL) or the healing caps (CL) full-arch pick-up impressions were taken with open impression trays loaded with elastomers (TurboFlex^®^, R&S, Paris, France) transferring screw-retained copings previously attached to implants and splinted between them by acrylic resin [24]. When the hybrid PEEK-NFC was planned for a single jaw, then the antagonist was recorded by an alginate impression. In the subsequent appointment, the validity of the master cast was checked by a passivity test with splinted titanium abutments that were screwed and explored by panoramic X-ray to assess the goodness of fit. If the fit was passive, then an intermaxillary occlusion record was taken at the proper vertical and centric relation following standard guidelines [25]. Otherwise, the structure was sectioned strategically and re-splinted until the passivity could be verified radiographically, being then transferred by a new pick-up impression. The next appointment assessed the occlusal and aesthetic composition by a milled PMMA test (Biostar^®^, Hinrichs, Goslar, Germany), to simulate the final restoration morphology (Figure 2). In most cases, the dental laboratory also used the tooth arrangement on either the interim fixed or the precedent prostheses as a starting point to manufacture this mockup test (Figure 2). After adjusting the occlusion on the mockup towards a mutually protected scheme, and determining the best color composition, the laboratory manufactured a replica by milling an infrastructure of PEEK from a disk (Smile-PEEK^®^, Pressing-Dental, San Marino, Italy) that were manually veneered by pink composite (Gradia Plus^®^, GC Ibérica, Madrid, Spain) on the gingival part of the rehabilitation and by multiple individual milled crowns made of the nanohybrid composite disc (Grandio^®^, VOCO GmbH, Cuxhaven, Germany) on the dental part. This final rehabilitation was definitively screwed onto implants to 20 Ncm.

### 2.4. Postoperative Assessments

One year after the final prostheses were delivered, the patient-centered outcomes (functional and subjective) were reassessed. Again, the masticatory performance estimated by mixing ability tests [20], the occlusal status recorded by Dental Prescale, and the muscular activity quantified by EMG were recorded as one-year functional outcomes. Similarly, the self-reported masticatory ability by the Leake Index [21], the impact on quality of life by OHIP-20 [19], and the self-reported oral satisfaction [22] were collected as subjective treatment outcomes.

However, the OHIP-20 questionnaire used in the preoperative phase was designed to capture the frequency of oral impacts over the 12 preceding months (OHIP-PRE), but in this postoperative phase two types of OHIP questionnaires were used: i.e., the OHIP-POST that refers to the oral impacts due to the prosthetic treatment, and the OHIP-THEN that recorded how patients think they were before treatment. All these designs are essential for estimating the true change in well-being. Theoretically, the patients who perceived improvements in OHQoL after treatment would obtain positive values after subtracting the total OHIP-POST score from either the total OHIP-PRE score (basic change) or the OHIP-THEN (alpha change), which is the least biased estimation of OHQoL change. Finally, a retrospective self-assessment of change among nine oral functions was collected by global transitional items (GTI) whose answers were Likert-type (from much worse to much better than before treatment).

#### Data Analysis

To assess the responsiveness of the OHIP-20 instrument we used the methodology proposed by other authors employing the OHIP [26], which detect several types of change (Alpha, Beta, and Gamma changes) [27] by using the aforementioned OHIP designs: Pre, before treatment; Post, after treatment; Then, performed after treatment but referring to baseline conditions. The magnitude of change was estimated by the effect size using the ranges proposed by Cohen: <0.2, no effect; 0.2–0.5, a slight effect; 0.5–0.8, a moderate effect; >0.8, a strong effect [28].

Chi Square Tests and McNemar Tests were used to compare nominal variables between groups and within groups, respectively. Similarly, Student *t*-tests and paired *t*-tests were carried out for intergroup and intragroup comparisons of quantitative variables, respectively. However, if data were not normally distributed, non-parametric tests (Mann–Whitney and Wilcoxon tests) were used instead of parametric ones for intergroup and within-groups comparisons, respectively.

Several stepwise linear regression models were calculated to find predictors of several patient-centered outcomes. The SPSS v.20 (Statistical Package for Social Sciences, Chicago, IL, USA) was used for all the statistical analyses establishing a *p*-value lower than 0.05 to declare a finding as statistically significant.

## 3. Results

### 3.1. Sample Description

As depicts Table 1, the sample was comprised of 34 adult patients aged, on average, 65.0 ± 10.0 years, from the low socio-occupational class (56%) that lived in Salamanca, Spain (62%), brushed their teeth at least once a day (80%), and currently did not smoke (70%). No significant differences were found between groups with respect to these sociodemographic and behavioral variables.

### 3.2. Baseline Anatomical Conditions

Table 2 shows that most implants were placed on type III quality in the maxilla (50.0%), and type II in the mandible (82%), mostly covered by medium–thick soft tissue in both jaws (88%). No significant differences between groups were found regarding these baseline anatomical parameters of the jaws.

### 3.3. Type of Rehabilitation and Distribution of Implant and Abutment Sizes

According to Table 3, half of the sample was treated in both jaws, 32.4% was treated only in the maxilla, and 17.6% only in the mandible. The antagonist was mostly fixed tooth-supported dentures or natural teeth (61.8%). On average, each patient was treated with 6.3 ± 2.3 implants, with 1.4 ± 1.3 implants placed in fresh extraction sockets and mature bone counterparts.

### 3.4. Change in the Occlusal Parameters after Prosthetic Treatments

Table 4 shows the PRESCALE parameters (i.e., contact area, average pressure, maximal pressure, and occlusal load) for both the full-arch and the anterior region, at baseline and one year after treatment. Intragroup comparisons by means of paired *t*-tests found significant differences between the preoperative total occlusal area (13.5 ± 10.6 mm^2^) and the total occlusal load (188.4 ± 201.0 Nw) one year after the PEEK-hybrid rehabilitation whose parameters increased to 31.6 ± 9.7 mm^2^ and 483.0 ± 2801 Nw, respectively. In addition, the maximal and average pressure significantly increased after treatment with the PEEK-hybrid fixed prostheses. Similar findings were observed for the anterior region and both sides.

Significant differences between loading groups were only observed in the postoperative full-arch recordings with regard to the average and maximal pressure, and their resulting occlusal load, which was significantly greater for those allocated to the IL group (606.5 ± 364.1 Nw) than among the CL group (398.2 ± 171.0 Nw).

### 3.5. Change of the EMG Muscular Activity after PEEK-Hybrid Rehabilitation

Table 5 depicts the EMG variations of both the masseter and temporalis muscles one year after rehabilitation with PEEK-Hybrid dentures. In the whole sample, a significant increment in the masseter activity with respect to baseline records was observed. This finding was also observed within the CL group. In all patients, the temporalis muscles showed lower bioelectrical activity in comparison with masseters muscles. A certain muscular symmetry was observed in both the preoperative and postoperative observations, although right-side recordings tended to be higher than counterparts.

In general, one year after treatment, the masseter activity increased from 26.6 ± 15.1 μv to 33.4 ± 16.4 μv on the right side, and from 23.5 ± 11.9 μv to 30.6 ± 14.5 μv on the left side (Table 5).

### 3.6. Change in Chewing Ability by Leake Index

The ability to chew estimated by the Leake Index [21] significantly increased one year after treatment with PEEK-hybrid dentures (Table 6). It was observed that one year after treatment, the difficulties in chewing carrots, salads, meat, boiled vegetables, and fresh apple significantly improved, although this change was greater for hard foods (carrot, meat, and apples) rather than for boiled vegetables. These findings were similar among the loading groups. The number of foods chewed without any difficulty changed from 1.0 ± 0.7 to 3.6 ± 1.9 pattern foods one year after treatment.

### 3.7. Change in Self-Rated Satisfaction One Year after Prosthetic Treatments

Table 7 shows that the rehabilitation with PEEK-Hybrid dentures significantly increased the global satisfaction (within the range from 4.2–5.5), the satisfaction with aesthetics (within the range from 4.0–5.7), and the satisfaction with mastication (within the range from 5.4–6.3). Moreover, the IL group reported significantly higher values of satisfaction than the CL group (Table 7) for all the types of satisfaction (global, aesthetic, chewing).

### 3.8. Change in Oral Health-Related Quality of Life after Prosthetic Treatments

According to the impact of OHQoL shown in Table 8, the baseline records indicate that the most affected dimensions were *pain* (3.2 ± 1.0; CI 95% = IC 95%: 2.8–3.5), *physical disability* (2.9 ± 1.4; IC 95%: 2.4–3.4), and *functional limitation* (2.5 ± 0.7; IC 95%: 2.2–2.7). After treatment with PEEK hybrid prostheses, the only domain with a certain impact (0.4 ± 0.7; IC 95%: 0.2–0.7) in both groups was *functional limitation* (2.0 ± 0.8). As depicted in Table 8, both groups afford comparable OHQoL in all domains, and after prosthetic rehabilitation with PEEK hybrid prostheses, significant improvements occurred across all domains, with the impact in OHQoL drastically reduced. According to the benchmarks proposed by Cohen [28], the effect of PEEK hybrid treatment on quality of life was very strong (effect size = 3.1 ± 1.0), with the greatest changes observed in the domains *pain* (effect size = 3.3 ± 1.1), *functional limitation* (effect size = 3.1 ± 1.2), *physical disability* (effect size = 2.1 ± 1.0), and *psychological discomfort* (effect size = 1.9 ± 1.0). In contrast, only minor or moderate effect occurs in the *social* (effect size = 0.6 ± 1.0) and *handicap* (effect size = 0.2 ± 1.3) dimensions, because the preoperative impacts were very low (Table 8).

### 3.9. Retrospective Self-Assessment of the Wellbeing Change by Global Transitional Items

Table 9 demonstrates that most patients perceived improvements after treatment across the nine domains assessed retrospectively by transitional items, although 11% of patients felt that oral hygiene had worsened after prostheses delivery. Major improvements were observed among mastication-related items, in which almost 90% of treated patients perceived their ability to chew and their feeding satisfaction as much better after treatment.

Both groups perceived comparable changes, although a significantly higher proportion of patients from the IL group (45.5%) perceived their *pronunciation* as much better after PEEK rehabilitation than counterparts (6.3%), Chi = 5.8; df: 2; *p* < 0.05.

### 3.10. Changes in Masticatory Performance by Mixing Ability Tests

No difference between groups regarding chewing performance assessed by mixing ability tests after PEEK hybrid treatments were found (Table 10). However, it was observed that mixing ability at ten chewing cycles significantly increases after treatment. Specifically, among the IL group, the masticatory performance increased from 15.7–31.5% recorded at baseline to 65.1–71.2% one year after treatment with full-arch hybrid rehabilitations. Similarly, among the CL group, the mixing ability changes from 15.0–23.3% to 63.4–71.7%.

### 3.11. Predictors of Patient-Centered Treatment Outcomes

Regarding mastication, the regression models summarized in Table 11 demonstrate that mixing ability is proportional to the number of easily chewed foods, i.e., each easily chewed food increased between 0.3 and 7.5 (CI 95%) the percentage of mixed fraction in the masticatory test. This model predicts the 14% of the variance of the masticatory performance (corrected R^2^ = 0.14). Regarding chewing ability, the regression model found that the number of easily chewed foods, captured by the Leake Index, depends on the masticatory performance at ten cycles (CI 95%: 0.01–0.09). This model was quite predictable (corrected R^2^ = 0.32). Focusing on patient feelings, the final global satisfaction depends exclusively on the loading cohort, with patients of the IL group more satisfied (CI 95%: 0.2–1.2) than the CL group, predicting 19% of the final satisfaction scores (corrected R^2^ = 0.19). Finally, the impact on OHQoL was proportional to the number of easily chewed foods of the Leake Index (CI 95%: 0.06–1.2; R^2^ = 0.14).

## 4. Discussion

The evidence regarding the impact of PEEK-Hybrid rehabilitations with IL is still scarce [15,16], supporting the relevancy of this RCT. Although in these aforementioned studies, the PEEK infrastructure was covered by acrylic resins instead of composite, the authors reported good clinical performance and adequate patient-reported outcomes in the short term.

This study found that despite both groups being comparable in terms of sociodemographic, behavioral, and anatomical variables, one year after treatment, the full occlusal area, the average pressure, and the total occlusal load was significantly higher among the IL group (Table 4). One way this difference could be explained is because the IL group had been trained with a fixed rehabilitation for a longer period than the CL group, whose provisional restoration was removable. In any case, the pre–post comparisons show that the occlusal area and the occlusal loading increase gradually from baseline conditions to fixed rehabilitation with PEEK-NFC (Table 4). Nevertheless, the present study found greater areas of occlusion than that reported by Montero [29] (26.1 ± 15.7 mm^2^); Baca et al. [30] (21.0 ± 10.9) mm^2^; Suzuki et al. [31] (10.3 ± 5.2) mm^2^; Iwaki [32] (7.0 ± 4.3) mm^2^, after using Dental PRESCALE in a comparable group of patients treated with two-implant overdentures [29,32] or conventional fixed rehabilitations [30,31]. These differences could be partially explained because of the distinct sensitivity of the pressure sheet (type 50 H, 97 μm thick) used in some studies [30,31,32] instead of the one used in the present study (MS type for medium pressure, 110 μm thick), but also because of the greater occlusal arrangement implemented by customized milled NFC crowns present on the present PEEK-NFC rehabilitations which is presumably better than that achieved when acrylic standardized teeth are used.

According to our findings, the final occlusal load (483.0 ± 280.1) N is lower than that reported by Baca [30] for patients treated with fixed porcelain-fused-to-metal rehabilitations (516.6 ± 85.6) N of 14 units but higher than that observed by the same author for patients treated with those rehabilitations distributed on 12 units (254.9 ± 116.4) N. In any case, the occlusal load of the PEEK-NFC is greater than that recorded by patients treated with two-implant retained overdentures (292.7 ± 163.2) N according to Montero et al. [29]; Baca (416.3 ± 137.2) N [30]; Suzuki [31] (342.1 ± 163.6) N; Iwaki [32] (157.9 ± 60.3) N. The Dental PRESCALE system can accurately measure occlusal forces within the range from 20–80 N, but their readings are usually greater than the actual load [33].

On the other hand, the effect of implant therapy on the muscular activity measured by electromyography (EMG) is rarely studied [34]. It has been recently estimated [34] that EMG recordings increased from 1.1–3.2 μV after treating edentulousness with either overdentures or fixed rehabilitations on implants, although, no significant difference was observed between either treatment alternatives. The same study reported a huge data disparity ranging between 58 and 320 μV for patients with either removable or fixed rehabilitations on implants, and between 66 and 520 μV for dentate control subjects.

In line with our results, Giannakopoulos et al. [35] also found great data dispersion with the EMG recorder (Noraxon^®^), higher activity on masseters in comparison with temporalis muscles, and a stable right muscular asymmetry. A balanced muscular activity may be an indicator of functional improvements of the masticatory system and may be even more relevant than the magnitude itself of the bioelectric tone of the elevators muscles, as reported by Montero et al. [29].

The present study found that treatment with fixed PEEK-NFC hybrid prostheses improves objectively mastication (Table 10), bite force, and occlusal pattern (Table 4). Moreover, patients perceived they could chew better (Table 6), and accordingly, were more satisfied with chewing (Table 7) and lower impact on the functional domain of OHQoL (Table 8 and Table 9) after treatment. Similar findings had been reported after implant rehabilitation with mandibular overdentures [29,30,31,32,33,34,35,36], but evidence from fixed hybrid rehabilitation is scarce [37,38], although, fixed rehabilitations are expected to be, at least, functionally equivalent to overdentures [39,40].

Focusing on satisfaction and quality of life, a clear improvement after prosthetic treatment was observed with satisfaction significantly higher among the IL than among the CL group (Table 7), but not in terms of quality of life (Table 8). In agreement with other studies [29,41,42] using the same assessment tools, the postoperative satisfaction and quality of life scores reported in the present study are higher than those observed among patients treated with two-implant retained overdentures. Specifically, the major improvements were observed among *pain*, *functional limitation*, and *physical disability* OHIP-dimensions (Table 8), as found in other studies [29,43]. In contrast, Kok failed to find significant differences between hybrids and overdentures regarding satisfaction and OHQoL in a randomized clinical trial [44]. Despite the relative consensus on the improvement in psychosocial and functional terms that implant fixed rehabilitations can offer to edentate patients in comparison with conventional complete dentures [38,39,40], or even compared to implant-retained overdentures [41], to date there is a need for studies assessing the impact of loading protocols on patient-based outcomes. Current evidence comes from CL implants in most studies, except for those studies focused on assessing the all-on-four concept in which all implants are immediately loaded with a provisional fixed prosthesis and then data from the CL group is missing [15,16]. In agreement with our results, some authors concluded that IL tends to improve the OHRQoL and satisfaction of mandibular overdenture wearers, faster and sooner than that observed with CL groups one year after implant treatments [29,43,45].

Finally, this study has demonstrated that the final impact on quality of life is linearly correlated with the number of pattern foods chewed without difficulty after treatment (Table 11), because *pain*, *physical disability*, and *functional limitation* are the most affected OHIP domains in the OHQoL of patients (Table 8), as reported elsewhere [46]. The loading protocol (IL vs. CL) was the only significant predictor for satisfaction in these regression models (Table 11). The levels of satisfaction reached by patients are similar to that reported in comparable short-term clinical trials [15,16]. Future studies should check the findings reported in the present study but with a larger sample size and longer follow-up periods.

In addition, in this study, the extent of PEEK rehabilitation (one or two arches) and the type of antagonist were not significant predictors of the major patient-centered outcomes (masticatory performance, foods easily chewed, global satisfaction, and final quality of life) according to these regression models (Table 1). This finding may be because, independently of the clinical baseline conditions, all patients were finally rehabilitated with fixed teeth in both arches (except three patients that maintained their comfortable metal-based removable partial dentures).

The patient-based outcomes reported in the present study depend mainly on the biomechanics of the prostheses (full-arch fixed dental prostheses) rather than on the material composition itself (PEEK-NFC) and that other options such as conventional metal-ceramic (metal framework veneered with hand-layered porcelain) or full ceramic restorations (veneered or monolithic frameworks) could get even better results. However, PEEK is a promising material because of its high biocompatibility, good mechanical properties, good wear resistance, low plaque affinity, and high bond strength with veneering composites/luting cements [47], although, few studies have assessed this material for CAD-CAM prostheses in clinical settings [13,14,15,16,47,48]. This material would be suitable for patients experiencing either metal or acrylic allergies and is cheaper than either conventional metal-ceramic or monolithic zirconia restorations. Clinical studies are needed to evaluate the long-term performance of these prostheses before PEEK can be safely recommended as an alternative to well-established prosthodontic material [47].

## 5. Conclusions

Treatment with PEEK-NFC hybrid prostheses significantly improves mastication, patient satisfaction, and oral health-related quality of life. The immediate loading increases the average and maximal occlusal bite force, as well as the final self-rated satisfaction.

## Figures and Tables

**Table 1:** Sociodemographic and behavioral description of the study sample (*n* = 34) and among implant groups. Comparisons between groups were made by means of Student *t*-test and Chi Square for quantitative and nominal variables, respectively.

Sociodemographic	All Patients (*n* = 34; 100%)		IL Group (*n* = 16; 47.1%)		CL Group (*n* = 18; 52.9%)	
Age Interval	*N*	%	*N*	%	*N*	%
40–59 yrs	8	23.5	5	21.3	3	16.7
60–70 yrs	17	50.0	7	43.8	10	55.6
>70 yrs	9	26.5	4	25.0	5	27.8
	Mean	SD	Mean	SD	Mean	SD
Age (yrs)	65.0	10.0	62.8	11.9	67.0	7.8
Gender	*N*	%	*N*	%	*N*	%
Female	15	44.1	7	43.8	8	44.4
Male	19	55.9	9	56.3	10	55.6
Socio-occupational class	*N*	%	*N*	%	*N*	%
Low	19	55.9	10	62.5	9	50.0
Medium	15	44.1	6	37.5	9	50.0
Place of residence	*N*	%	*N*	%	*N*	%
Urban	21	61.8	10	62.5	11	61.1
Peri-urban	3	8.8	2	12.5	1	5.6
Rural	10	29.4	4	25.0	6	33.3
Behavioral						
Brushing habits	*N*	%	*N*	%	*N*	%
2–3 times a day	7	20.6	3	18.8	4	22.2
Once a day	16	47.1	6	37.5	10	55.6
Less than once a day	11	32.4	7	43.8	4	22.2
Smoking habits	*N*	%	*N*	%	*N*	%
Non-smoker	17	50.0	7	43.8	10	55.6
Current smoker	10	29.4	5	31.2	5	27.8
Past Smoker	7	20.6	4	25.0	3	16.6
	Mean	Sd	Mean	Sd	Mean	Sd
Cigarettes/day in smokers	13.5	5.3	12.8	5.1	14.3	5.9

**Table 2:** Anatomical conditions of the jaws of the study sample (*n* = 34) at baseline. Comparisons by Chi Square and Student *t*-tests.

	All Patients (*n* = 34; 100%)		IL Group (*n* = 16; 47.1%)		CL Group (*n* = 18; 52.9%)	
Bone Quality in Maxilla according to Leckholm and Zarb (*n* = 28 patients)						
	*N*	%	*N*	%	*N*	%
Type I	0	0.0	0	0.0	0	0.0
Type II	2	7.1	1	7.1	1	7.1
Intermediate	8	28.6	7	50.0	1	7.1
Type III	11	39.3	4	28.6	7	50.0
Intermediate	3	10.7	1	7.1	2	14.3
Type IV	4	14.3	1	7.1	3	21.4
Total	28	100.0	14	100.0	18	100
Bone Quality in the mandible according to Leckholm and Zarb (*n* = 22 patients)						
Type I	1	4.5	0	0.0	1	9.1
Type II	8	36.4	4	36.4	4	36.4
Intermediate	10	45.5	5	45.5	5	45.5
Type III	3	13.6	2	18.2	1	9.1
Intermediate	0	0.0	0	0.0	0	0.0
Type IV	0	0.0	0	0.0	0	0.0
Total	22	100	11	100	11	100
Gingival Biotype in MAXILLA						
Gingival Biotype	*N*	%	*N*	%	*N*	%
fine	0	0.0	0	0.0	0	0.0
medium	14	50.0	7	50.0	7	50.0
thick	14	50.0	7	50.0	7	50.0
Gingival Biotype in MANDIBLE						
Gingival Biotype	*N*	%	*N*	%	*N*	%
fine	5	22.7	3	27.3	2	18.2
medium	14	63.6	6	54.5	8	72.7
thick	3	13.6	2	18.2	1	9.1
	Mean	Sd	Mean	Sd	Mean	Sd

**Table 3:** Distribution of the type of rehabilitation among patients and number of implants per patient (*n* = 34).

Implant-Related Variables	All Patients (*n* = 34 Patients with 210 Implants)		IL Group (*n* = 16 Patients with 96 Implants)		CL Group (*n* = 18 Patients with 114 Implants)	
	*N*	%	*N*	%	*N*	%
Type of hybrid PEEK-NFC Rehabilitation						
Lower PEEK	6	17.6	2	12.5	4	22.2
Upper PEEK	11	32.4	5	31.3	6	33.3
Bimaxilar PEEK	17	50.0	9	56.3	8	44.4
Type of Antagonist						
Partial Denture	3	8.8	0	0.0	3	16.7
Fixed implant-supported prosthesis	10	29.4	4	25.0	6	33.3
Fixed tooth-supported denture/Natural	21	61.8	12	75.0	9	50.0
Number of Implants	Mean	Sd	Mean	Sd	Mean	Sd
Total	6.3	2.3	6.8	2.5	5.8	2.1
Postextractive implants	1.4	1.3	1.8	1.3	1.1	1.2
	N	%	N	%	N	%

**Table 4:** Changes in the PRESCALE occlusal parameters one year after rehabilitation with PEEK-NFC (*n* = 34).

Baseline Records	All Patients (*n* = 34 Patients at Baseline; *n* = 27 at Follow-Up)		IL Group (*n* = 16 at Baseline; *n* = 11 at Follow-Up)		CL Group (*n* = 18 Patients at Baseline; *n* = 16 at Follow-Up)	
Full Arch	Mean	SD	Mean	SD	Mean	SD
Contact Area (mm)	13.5 ^a^	10.6	13.6 ^a^	9.8	13.4 ^a^	11.5
Average Pressure (MPa)	12.2 ^a^	13.7	13.0 ^a^	4.8	11.5 ^a^	2.4
Maximal Pressure (MPa)	35.4 ^a^	7.1	36.6 ^a^	9.4	34.3 ^a^	4.2
Occlusal Load (Nw)	188.4 ^a^	201.0	224.6 ^a^	256.8	156.2 ^a^	133.8
Anterior Region	Mean	SD	Mean	SD	Mean	SD
Contact Area (mm)	6.3 ^a^	4.4	6.0 ^a^	4.1	6.5 ^a^	4.7
Average Pressure (MPa)	11.1 ^a^	3.0	11.5 ^a^	3.2	10.7 ^a^	2.9
Maximal Pressure (MPa)	31.9 ^a^	11.5	33.7 ^a^	13.9	30.3 ^a^	9.0
Occlusal Load (Nw)	80.2 ^a^	73.3	79.6 ^a^	89.1	80.7 ^a^	58.5
Right Side	Mean	SD	Mean	SD	Mean	SD
Contact Area (mm)	4.2 ^a^	4.3	4.3 ^a^	4.6	4.2 ^a^	4.1
Average Pressure (MPa)	9.9 ^a^	4.2	10.7 ^a^	5.6	9.2 ^a^	2.4
Maximal Pressure (MPa)	25.4 ^a^	11.5	27.1 ^a^	13.4	23.9 ^a^	9.6
Occlusal Load (Nw)	50.4 ^a^	56.2	56.8 ^a^	65.1	44.7 ^a^	48.1
Left Side	Mean	SD	Mean	SD	Mean	SD
Contact Area (mm)	4.1 ^a^	4.1	4.7 ^a^	5.0	3.6 ^a^	3.0
Average Pressure (MPa)	10.0 ^a^	^a^ 3.3	9.9 ^a^	4.2	10.0 ^a^	2.5
Maximal Pressure (MPa)	25.3 ^a^	11.0	27.4 ^a^	12.3	23.4 ^a^	9.8
Occlusal Load (Nw)	45.4 ^a^	43.3	52.4 ^a^	52.7	39.1	33.2
Postoperative Records (One Year After Treatment)						
Full-Arch	Mean	SD	Mean	SD	Mean	SD
Contact Area (mm)	31.6 ^b^	9.7	35.2 ^b^	9.3	29.1 ^b^	9.4
Average Pressure (MPa) *	15.4 ^b^	4.2	18.3 ^b^	4.5	13.4 ^b^	2.3
Maximal Pressure (MPa) *	42.9 ^b^	10.7	47.4 ^b^	13.9	39.8 ^b^	6.7
Occlusal Load (Nw) *	483.0 ^b^	280.1	606.5 ^b^	364.1	398.2 ^b^	171.0
Anterior Region						
Contact Area (mm)	10.2 ^b^	7.8	9.6 ^b^	7.3	10.6 ^b^	8.4
Average Pressure (MPa)	13.0 ^b^	4.6	14.2 ^a^	6.4	12.2 ^a^	2.8
Maximal Pressure (MPa)	37.9 ^a^	22.0	43.3 ^a^	31.0	34.1 ^a^	12.6
Occlusal Load (Nw)	160.0 ^b^	174.5	182.0 ^b^	245.3	144.8 ^b^	109.8
Right Side	Mean	SD	Mean	SD	Mean	SD
Contact Area (mm)	13.9 ^b^	4.3	15.3 ^b^	4.9	12.9 ^b^	3.7
Average Pressure (MPa)	15.0 ^b^	4.3	15.7 ^a^	3.6	14.4 ^b^	4.7
Maximal Pressure (MPa)	36.7 ^b^	7.8	39.6 ^b^	8.2	34.6 ^b^	7.1
Occlusal Load (Nw)	207.9 ^b^	89.6	244.1 ^b^	104.6	182.9 ^b^	70.7
Left Side	Mean	SD	Mean	SD	Mean	SD
Contact Area (mm)	14.4 ^b^	5.5	15.6 ^b^	6.4	13.6 ^b^	4.9
Average Pressure (MPa)	15.1 ^b^	4.1	15.8 ^b^	4.4	14.6 ^b^	3.9
Maximal Pressure (MPa)	38.6 ^b^	12.2	42.9 ^b^	16.8	35.7 ^b^	6.9
Occlusal Load (Nw)	201.4 ^b^	110.2	249.0 ^b^	144.8	194.1 ^b^	75.9

^a,b^ Lower-case distinct letters within the columns mean significant pre-post differences (*p* < 0.05) after paired *t*-test, which have the preoperative values as reference. * Significant differences *p* < 0.5 between groups after Student *t*-tests.

**Table 5:** Changes in the muscular electromyographic records (μv) after PEEK-Hybrid prosthetic treatments (*n* = 34 at baseline; *n* = 27 at follow-up).

EMG Maximal Force (μv)	All Patients (*n* = 34 Patients at Baseline; *n* = 27 at Follow-Up)		IL Group (*n* = 16 at Baseline; *n* = 11 at Follow-Up)		CL Group (*n* = 18 Patients at Baseline; *n* = 16 at Follow-Up)	
Preoperative	Mean	SD	Mean	SD	Mean	SD
Masseter Right	26.6 ^a^	15.1	29.8 ^a^	18.6	23.8 ^a^	10.9
Masseter Left	23.5 ^a^	11.9	24.3 ^a^	13.4	22.8 ^a^	10.7
Temporal Right	22.9 ^a^	14.0	22.6 ^a^	14.2	23.3 ^a^	14.3
Temporal Left	21.7 ^a^	13.1	19.5 ^a^	10.3	23.6 ^a^	15.2
Postoperative						
Masseter Right	33.4 ^b^	16.4	36.4 ^a^	19.8	31.3 ^b^	13.9
Masseter Left	30.6 ^b^	14.5	33.6 ^b^	16.3	28.5 ^b^	13.3
Temporal Right	24.9 ^a^	14.3	24.9 ^a^	16.0	24.9 ^a^	13.6
Temporal Left	22.7 ^a^	11.3	21.8 ^a^	7.8	23.3 ^a^	13.4

^a,b^ Lower-case distinct letters within the columns mean significant differences (*p* < 0.05) after paired *t*-test, which have the preoperative values as reference.

**Table 6:** Changes in the ability to chew according to Leake Index one year after PEEK-Hybrid dentures (*n* = 34 at baseline; *n* = 27 at follow-up).

All Patients	Carrot		Salads		Meat		Vegetables		Apple		Number of Foods Easily Chewed	
Baseline ^a^	*N*	%	*N*	%	*N*	%	*N*	%	*N*	%	Mean	SD
Easy	0	0.0	6	17.6	1	2.9	28	82.4	0	0.0	1.0 ^a^	0.7
A bit difficult	4	11.8	19	55.9	14	41.2	6	17.6	6	17.6		
Very difficult	30	88.2	9	26.5	19	55.9	0	0.0	28	82.4		
12 months after implant hybrid PEEK ^b^												
Easy	23	85.2	27	100.0	26	96.3	27	100.0	18	66.7	3.6 ^b^	1.9
A bit difficult	4	14.8	0	0.0	1	3.7	0	0.0	9	33.3		
Very difficult	0	0.0	0	0.0	0	0.0	0	0.0	0	0.0		
IL Group												
Baseline ^a^	*N*	%	*N*	%	*N*	%	*N*	%	*N*	%	Mean	SD
Easy	0	0.0	2	12.5	1	6.3	14	87.5	0	0.0	1.1 ^a^	0.7
A bit difficult	3	18.8	11	68.8	6	37.5	2	12.5	4	25.0		
Very difficult	13	81.3	3	18.8	9	56.3	0	0.0	12	75.0		
12 months after implant hybrid PEEK ^b^												
Easy	11	100.0	11	100.0	10	90.9	11	100.0	9	81.8	3.3 ^b^	2.3
A bit difficult	0	0.0	0	0.0	1	9.1	0	0.0	2	18.2		
Very difficult	0	0.0	0	0.0	0	0.0	0	0.0	0	0.0		
CL Group												
Baseline ^a^	*N*	%	*N*	%	*N*	%	*N*	%	*N*	%	Mean	SD
Easy	0	0	4	22.2	0	0.0	14	77.8	0	0.0	1.0 ^a^	0.7
A bit difficult	1	5.6	8	44.4	8	44.4	4	22.2	2	11.1		
Very difficult	17	94.4	6	33.3	10	55.6	0	0.0	16	88.9		
12 months after implant hybrid PEEK ^b^												
Easy	12	75.0	16	100.0	16	100.0	16	100.0	9	56.3	3.8 ^b^	1.4
A bit difficult	4	25.0	0	0.0	0	0.0	0	0.0	7	43.7		
Very difficult	0	0.0	0	0.0	0	0.0	0	0.0	0	0.0		

^a,b^ Lower-case distinct letters within the columns mean significant differences (*p* < 0.05) after paired *t*-test or McNemar Test, which have the preoperative values as reference.

**Table 7:** Changes in self-rated chewing, aesthetic, and global satisfaction on a 0–10 scale after prosthetic treatments (*n* = 34 at baseline; *n* = 27 at follow-up).

Satisfaction (0–10 Range)	All Patients (*n* = 34 Patients at Baseline; *n* = 27 at Follow-Up)		IL Group (*n* = 16 at Baseline; *n* = 11 at Follow-Up)		CL Group (*n* = 18 Patients at Baseline; *n* = 16 at Follow-Up)	
Preoperative	Mean	SD	Mean	SD	Mean	SD
Global	4.2 ^a^	1.5	3.9 ^a^	1.5	4.4 ^a^	1.4
Aesthetic	4.3 ^a^	1.7	4.2 ^a^	2.0	4.4 ^a^	1.5
Chewing	3.4 ^a^	1.1	3.1 ^a^	1.0	3.6 ^a^	1.2
Postoperative						
Global *	9.1 ^b^	0.7	9.5 ^b^	0.6	8.8 ^b^	0.7
Aesthetic *	9.0 ^b^	1.1	9.5 ^b^	0.7	8.7 ^b^	1.2
Chewing *	9.1 ^b^	0.5	9.6 ^b^	0.7	9.1 ^b^	0.5

^a,b^ Lower-case distinct letters within the columns mean significant pre-post differences (*p* < 0.05) after paired *t*-test, which have the preoperative values as reference. * Significant differences *p* < 0.5 between groups after Student *t*-tests.

**Table 8:** Changes in oral health-related quality of life (OHIP-20), by simple count-method ^φ^ after prosthetic treatments (*n* = 34 at baseline; *n* = 27 at follow-up).

Oral Health-Related Quality of Life OHIP-20	All Patients (*n* = 34 Patients at Baseline; *n* = 27 at Follow-Up)		IL Group (*n* = 16 at Baseline; *n* = 11 at Follow-Up)		CL Group (*n* = 18 Patients at Baseline; *n* = 16 at Follow-Up)	
Preoperative Scores	Mean	SD	Mean	SD	Mean	SD
Functional limitation	2.5 ^a^	0.7	2.5 ^a^	0.7	2.4 ^a^	0.7
Pain	3.2 ^a^	1.0	3.1 ^a^	1.1	3.2 ^a^	0.9
Psychological Discomfort	1.5 ^a^	0.8	1.6 ^a^	0.7	1.4 ^a^	0.8
Physical Disability	2.9 ^a^	1.4	3.0 ^a^	1.5	2.9 ^a^	1.5
Psychological Disability	1.3 ^a^	0.9	1.6 ^a^	0.8	1.0 ^a^	0.9
Social Disability	0.7 ^a^	0.9	0.8 ^a^	0.9	0.6 ^a^	0.9
Handicap	0.1 ^a^	0.4	0.1 ^a^	0.5	0.1 ^a^	0.2
Total	12.1 ^a^	3.7	12.6 ^a^	3.9	11.6 ^a^	3.6
Postoperative Scores	Mean	SD	Mean	SD	Mean	SD
Functional limitation	0.4 ^b^	0.7	0.4 ^b^	0.7	0.5 ^b^	0.7
Pain	0.0 ^b^	0.2	0.0 ^b^	0.0	0.1 ^b^	0.2
Psychological Discomfort	0.0 ^b^	0.2	0.0 ^b^	0.0	0.1 ^b^	0.3
Physical Disability	0.0 ^b^	0.0	0.0 ^b^	0.0	0.0 ^b^	0.0
Psychological Disability	0.1 ^b^	0.3	0.1 ^b^	0.3	0.1 ^b^	0.3
Social Disability	0.0 ^b^	0.0	0.0 ^b^	0.0	0.0 ^b^	0.0
Handicap	0.1 ^b^	0.3	0.0 ^b^	0.0	0.0 ^b^	0.2
Total	0.6 ^b^	1.0	0.6 ^b^	0.9	0.7 ^b^	1.1
Effect Sizes	Mean	SD	Mean	SD	Mean	SD
Functional limitation	3.1	1.2	3.5	1.0	2.8	1.3
Pain	3.3	1.1	3.2	1.1	3.3	1.0
Psychological Discomfort	1.9	1.0	2.2	0.9	1.8	1.1
Physical Disability	2.1	1.0	2.2	1.0	2.0	1.1
Psychological Disability	1.2	1.0	1.6	0.9	0.9	1.0
Social Disability	0.6	1.0	0.7	1.0	0.5	1.0
Handicap	0.2	1.3	0.2	1.8	0.2	0.7
Total	3.1	1.0	3.4	1.0	2.9	1.0

^φ^ The presence of any impact among items was recorded as present if it was reported at the threshold of “occasional” or more frequently. The number of impacts per person was calculated by the simple counting of items with impact across domains. ^a,b^ Lower-case distinct letters mean very significant differences (*p* < 0.01) after paired *t*-test, taking the preoperative values as reference.

**Table 9:** Retrospective evaluation of the prosthetic treatments (Hybrid Full-Arch PEEK-NFC) by Global Transition Items in the following sample (*n* = 27) and within loading groups.

Effect of Prosthetic Treatment	Much Worse	Worse	Equal	Better	Much Better
All Patients (*n* = 27 at follow-up)	*N* (%)	*N* (%)	*N* (%)	*N* (%)	*N* (%)
Pronouncing words *	0 (0.0)	0 (0.0)	18 (66.7)	3 (11.1)	6 (22.2)
Taste and smell	0 (0.0)	0 (0.0)	12 (44.4)	11 (40.7)	4 (14.8)
Painful aching in the mouth	0 (0.0)	0 (0.0)	1 (3.7)	16 (59.3)	10 (37.0)
Oral hygiene	0 (0.0)	3 (11.1)	5 (18.5)	14 (59.1)	5 (18.5)
Chewing Ability	0 (0.0)	0 (0.0)	0 (0.0)	2 (7.4)	25 (92.6)
Feeding satisfaction	0 (0.0)	0 (0.0)	0 (0.0)	3 (11.1)	24 (88.9)
Mouth comfortability	0 (0.0)	1 (3.7)	0 (0.0)	7 (25.9)	19 (70.4)
Appealing Smile	0 (0.0)	0 (0.0)	0 (0.0)	9 (33.3)	18 (66.7)
Social relations	0 (0.0)	0 (0.0)	8 (29.6)	16 (59.3)	3 (11.1)
IL Group (*n* = 11 at follow-up)	*N* (%)	*N* (%)	*N* (%)	*N* (%)	*N* (%)
Pronouncing words	0 (0.0)	0 (0.0)	5 (45.5)	1 (9.1)	5 (45.5)
Taste and smell	0 (0.0)	0 (0.0)	4 (36.4)	4 (36.4)	3 (27.3)
Painful aching in the mouth	0 (0.0)	0 (0.0)	0 (0.0)	4 (36.4)	7 (63.6)
Oral hygiene	0 (0.0)	0 (0.0)	2 (18.2)	7 (63.6)	2 (18.2)
Chewing Ability	0 (0.0)	0 (0.0)	0 (0.0)	0 (0.0)	11 (100)
Feeding satisfaction	0 (0.0)	0 (0.0)	0 (0.0)	1 (9.1)	10 (90.9)
Mouth comfortability	0 (0.0)	0 (0.0)	0 (0.0)	2 (18.2)	9 (81.8)
Appealing Smile	0 (0.0)	0 (0.0)	0 (0.0)	2 (18.2)	9 (81.8)
Social relations	0 (0.0)	0 (0.0)	2 (18.2)	8 (72.7)	1 (9.1)
CL Group (*n* = 16 at follow-up)	*N* (%)	*N* (%)	*N* (%)	*N* (%)	*N* (%)
Pronouncing words	0 (0.0)	0 (0.0)	13 (81.3)	2 (12.5)	1 (6.3)
Taste and smell	0 (0.0)	0 (0.0)	8 (50.0)	7 (43.8)	1 (6.3)
Painful aching in the mouth	0 (0.0)	0 (0.0)	1 (6.3)	12 (75.0)	3 (18.8)
Oral hygiene	0 (0.0)	3 (18.8)	3 (18.8)	7 (43.8)	3 (18.8)
Chewing Ability	0 (0.0)	0 (0.0)	0 (0.0)	2 (12.5)	14 (87.5)
Feeding satisfaction	0 (0.0)	0 (0.0)	0 (0.0)	2 (12.5)	14 (87.5)
Mouth comfortability	0 (0.0)	1 (6.3)	0 (0.0)	5 (31.3)	10 (62.5)
Appealing Smile	0 (0.0)	0 (0.0)	0 (0.0)	7 (43.8)	9 (56.3)
Social relations	0 (0.0)	0 (0.0)	6 (37.5)	8 (50.0)	2 (12.5)

* Significant differences *p* < 0.5 between groups after Chi Square Tests.

**Table 10:** Changes in masticatory performance at ten chewing strokes assessed by mixing ability test (ChewingApp) of bicolored chewing-gum (*n* = 34 at baseline; *n* = 27 at follow-up).

Masticatory Performance by Mixing Ability Tests (https://studio.chewing.app/)	All Patients (*n* = 34 Patients at Baseline; *n* = 27 at Follow-Up)			IL Group (*n* = 16 at Baseline; *n* = 11 at Follow-Up)		CL Group (*n* = 18 Patients at Baseline; *n* = 16 at Follow-Up)
	Mean	SD	Mean	SD	Mean	SD
Preoperative Scores	19.7 ^a^	9.0	21.0 ^a^	10.6	18.6 ^a^	7.6
Postoperative Scores	67.8 ^b^	6.5	68.2 ^b^	4.6	67.5 ^b^	7.7

^a,b^ Lower-case distinct letters within the columns mean significant differences (*p* < 0.05) after paired *t*-test, taking the preoperative as reference.

**Table 11:** Linear Regression Analyses for predicting the treatment outcomes as a function of the age, sex, cohort, extent of rehabilitation (one or both arches), type of antagonist, bone quality, final masticatory performance, final chewing ability, satisfaction, quality of life, final occlusal area, final occlusal load, and muscular activity (*n* = 34).

Dependent Predictors	Β	Error	T	*p*-Value	Lower CI 95%	Upper CI 95%
Masticatory Performance ^a^						
Postoperative easily chewed foods	3.9	0.03	2.3	0.03	0.34	7.52
Foods Chewed Easily ^b^						
Postoperative masticatory performance	0.05	0.02	2.8	0.01	0.01	0.09
Global Satisfaction ^c^						
Loading Cohort	0.67	0.26	2.6	0.02	0.2	1.2
Final Impact on Quality of Life ^d^						
Foods easily chewed after treatment	−0.63	0.28	−2.3	0.03	−0.06	−1.2

^a^ F = 5.1; *p* < 0.05. Corrected R^2^ = 0.14. ^b^ F = 6.8; *p* < 0.01. Corrected R^2^ = 0.32. ^c^ F = 6.9; *p* < 0.05. Corrected R^2^ = 0.19. ^d^ F = 5.2; *p* < 0.05. Corrected R^2^ = 0.14.
